# Prolonged Helium Postconditioning Protocols during Early Reperfusion Do Not Induce Cardioprotection in the Rat Heart *In Vivo*: Role of Inflammatory Cytokines

**DOI:** 10.1155/2015/216798

**Published:** 2015-01-27

**Authors:** Gezina Tanya Mei Ling Oei, Hamid Aslami, Raphaela Priscilla Kerindongo, Renske Johanna Steenstra, Charlotte Jacqueline Peter Beurskens, Anita Maria Tuip-de Boer, Nicole Petra Juffermans, Markus Werner Hollmann, Benedikt Preckel, Nina Claudia Weber

**Affiliations:** ^1^Laboratory of Experimental Anesthesiology and Intensive Care, Department of Anesthesiology, Academic Medical Centre, Meibergdreef 9, 1100 DD Amsterdam, Netherlands; ^2^Laboratory of Experimental Anesthesiology and Intensive Care, Department of Intensive Care, Academic Medical Centre, Meibergdreef 9, 1100 DD Amsterdam, Netherlands

## Abstract

Postconditioning of myocardial tissue employs short cycles of ischemia or pharmacologic agents during early reperfusion. Effects of helium postconditioning protocols on infarct size and the ischemia/reperfusion-induced immune response were investigated by measurement of protein and mRNA levels of proinflammatory cytokines. Rats were anesthetized with S-ketamine (150 mg/kg) and diazepam (1.5 mg/kg). Regional myocardial ischemia/reperfusion was induced; additional groups inhaled 15, 30, or 60 min of 70% helium during reperfusion. Fifteen minutes of helium reduced infarct size from 43% in control to 21%, whereas 30 and 60 minutes of helium inhalation led to an infarct size of 47% and 39%, respectively. Increased protein levels of cytokine-induced neutrophil chemoattractant (CINC-3) and interleukin-1 beta (IL-1*β*) were found after 30 or 60 min of helium inhalation, in comparison to control. 30 min of helium increased mRNA levels of CINC-3, IL-1*β*, interleukin 6 (IL-6), and tumor necrosis factor alpha (TNF-*α*) in myocardial tissue not directly subjected to ischemia/reperfusion. These results suggest that the effectiveness of the helium postconditioning protocol is very sensitive to duration of noble gas application. Additionally, helium was associated with higher levels of inflammatory cytokines; however, it is not clear whether this is causative of nature or part of an epiphenomenon.

## 1. Introduction

Early reperfusion is the cornerstone in the treatment of myocardial infarction, as quick restoration of blood flow to compromised areas will reduce infarct size, hence reducing the development of heart failure, myocardial stunning, and arrhythmias [1]. The flipside of the coin is reperfusion injury: cellular dysfunction that is caused by the hyperacute return of blood flow and oxygen to the tissue. The primary mechanisms of reperfusion-induced cell damage are oxidative stress and an inflammatory burst [2]. The mechanism of reperfusion injury suggests that any therapeutic intervention aiming to reduce this reperfusion-induced injury should be effective at the time of opening of the (partially) obstructed vessel. It resulted in the discovery of pre- [3] and postconditioning [4], techniques reducing infarct size by applying repetitive, short cycles of ischemia before or after the ischemic insult and exerting its effects primarily at the time point of reperfusion.

“Conditioning” can also be achieved by application of volatile anesthetics (sevoflurane, isoflurane) or noble gases (xenon, helium) according to specific protocols [5]. In rats, inhalation of  15 minutes of  70% helium before or immediately after 25 minutes of myocardial ischemia reduced infarct size after 120 minutes of reperfusion [6, 7]. Currently it is unknown whether the duration of the helium postconditioning protocol influences infarct size after myocardial regional I/R. Considering future application of helium postconditioning in patients, it is relevant to know whether the length of exposure affects the outcome in a positive or negative way.

A mechanism for helium-induced cardioprotection has yet not been elucidated, although several potential pathways have been suggested, such as signaling kinases of the RISK-pathway and the mitochondrial permeability transition pore [8]. The role of the innate immune system in I/R injury has been under debate for years, as an excessive inflammatory burst is detrimental but at the same time a general inhibition of the innate immune system is associated with adverse outcome after myocardial infarction [9]. During early reperfusion, several processes occur such as leukocyte activation and recruitment, cytokine and reactive oxygen species burst, and concomitant endothelial dysfunction [10]. Taken together, stimulation of a sterile immune response during acute onset of reperfusion influences cell viability.

Currently, we do not know whether helium postconditioning affects the cytokine burst during early reperfusion. Therefore, in this study we exposed rats undergoing regional I/R to various lengths of helium postconditioning protocols and assessed myocardial damage. We hypothesized that helium-induced cardioprotection is accompanied by a reduction in the sterile immune response during reperfusion. We, therefore, analyzed CINC-3, IL-6, IL-1*β*, and TNF-*α* at protein level and at mRNA levels in myocardium exposed to I/R (“area at risk”, AAR) and myocardium not downstream from the occluded coronary artery (“area not at risk”, NAAR).

## 2. Materials and Methods

### 2.1. Rat Model of Regional Myocardial I/R

Helium 70%/oxygen 30% was purchased from Linde Gas (Linde Gas Benelux BV, Dieren, The Netherlands). Male adult Wistar rats (Charles River, Wilmington, MA, USA) were acclimatized for 7 days under conditions of 12 h light and dark cycles and had free access to food and water. Animals were treated in accordance with the Guide for the Care and Use of Laboratory Animals (NIH Publication number 85-23, revised 1996) and all experiments were approved by the institute's animal ethics committee (DAA102650 and 102279). Surgery of the animals was performed as previously described [11] and comprised reversible ligation of a great branch of the left descending coronary artery. All animals were connected to blood pressure and heart frequency monitoring by cannulation of the carotid artery and had a 15-minute baseline period for stabilization after surgical preparation. Additionally, animals were connected to a mechanical ventilator and inhaled 30% oxygen throughout the experimental protocol, which was verified by using an oxygen sensor on the ventilator indicating the percentage of inspired oxygen and by randomly performing blood gas analysis.

### 2.2. Study Design

For investigation of the effect of various helium postconditioning protocols on cellular damage within the myocardium we divided animals in 5 groups. Sham operated animals (Sham) underwent surgery but did not undergo ischemia and reperfusion. The control group (CON) underwent 25 minutes of ischemia and 120 minutes of reperfusion. The helium intervention groups also underwent 25 minutes of ischemia and 120 minutes of reperfusion but additionally inhaled 15, 30, or 60 minutes of 70% helium/30% oxygen at the onset of reperfusion (He15, He30, and He60). To make sure adequate helium concentrations were present at the onset of reperfusion, inhalation of helium was started in the last minute of the ischemic episode. Cellular damage was investigated by infarct size staining of myocardial slices and measurement of serum levels of lactate dehydrogenase (LDH) and troponine-T (TnT). For infarct size measurements, the heart was excised under deep anesthesia and treated for further analysis as described previously [6].

Similar experiments were conducted to obtain myocardial tissue for the measurement of protein levels of cytokines TNF-*α*, IL-1*β*, IL-6, and chemokine CINC-3. At the end of the experimental protocol (i.e., after 15 min of stabilization plus 25 min of ischemia plus 120 min of reperfusion), hearts were excised and kept at −80°C until further processing for ELISA measurements. Messenger RNA levels of the proinflammatory markers TNF-*α*, IL-1*β*, IL-6, and chemokine CINC-3 were investigated in experiments with short reperfusion protocols. For this purpose, rats were assigned to 7 groups. We assumed that in our model, in accordance with any biological system, transcription of mRNA precedes translation and upregulation of a protein. If any effect of helium postconditioning on mRNA level exists, it should be found in the hyperacute phase after the intervention (before profound effects on protein level can be found). Therefore, myocardial tissue for mRNA analyses was harvested immediately after the specific reperfusion episode. As it is already shown that 15 minutes of helium reduces infarct size [6, 7], it is crucial to find out if any transcriptional effects can be found as early as after 5 minutes of helium exposure. Additionally it should be investigated whether prolonged exposure results in profound up- or downregulation of inflammatory mRNA. Three groups of animals underwent 25 minutes of ischemia and 5, 15, or 30 minutes of reperfusion, respectively (I/R5, I/R15, and I/R30). The helium intervention groups underwent 25 minutes of ischemia and 5, 15, or 30 minutes of reperfusion with simultaneous inhalation of helium during the entire reperfusion episode. After completion of the experimental protocol, the duration varied in each group, hearts were excised and kept at −80°C until further processing for RT-PCR experiments.

For investigation of protein and mRNA levels of inflammatory markers, we distinguished two types of myocardial (ventricular) tissue: area at risk (AAR, ischemic tissue) and area not at risk (NAAR, nonischemic tissue). After excision of the whole heart, ventricular tissue was separated from the atrial tissue and removed from valves and remains of great vessels. As the (opened) ligature was left in situ, we could clearly point out the area of the ventricle downstream of the ligature: the AAR. In all groups except the Sham group, the distinct color of the AAR in comparison to the surrounding tissue further enabled separate excision. This part of the ventricle was cut from the rest of the ventricle on ice and comprised approximately one-quarter to one-fifth of total ventricular tissue. At the opposite side of the ventricle, not downstream of the ligature, we excised a second part of the ventricular tissue: the “area not at risk” (NAAR).

### 2.3. Infarct Size Determination and Measurement of LDH and TnT

The area at risk and the infarcted area were determined by planimetry using SigmaScan Pro 5 software (SPSS Science Software, Chicago, IL, USA). For measurement of injury markers LDH and TnT, arterial blood sampling through the carotid cannula was done at baseline, after 24 minutes of ischemia, after application of the conditioning stimulus, and after 120 minutes of reperfusion. After sampling, blood was centrifuged at 3100 RPM, 20°C for 10 min. Serum was kept at −80°C until measurement of LDH and TnT by ELISA in our clinical laboratory.

### 2.4. Homogenization and Fractionation

After separation of the AAR from the NAAR on ice, tissue was subsequently snap frozen in liquid nitrogen for storage at −80°C until further analysis. Hearts were weighted and accordingly diluted with normal saline (1 mg of heart tissue was multiplied by 4, resulting in the amount of saline in mL to be added) and Greenberger Lysis buffer (105 mM NaCl, 15 mM Tris, 1 mM MgCl·H_2_O, 1 mM CaCl_2_, 1% Triton, and destilled water) with protease inhibitor mix (pepstation A, leupeptin, aprotinin) (1 : 5) upon homogenization. After homogenization, samples were left on ice for 20 minutes and subsequently centrifuged for 10 minutes at 3600 RPM at 4°C. Supernatants were stored at −80°C for ELISA analysis. For qRT-PCR analysis, homogenates were stored with Tripure isolation reagent (1 : 9) at −80°C.

### 2.5. Enzyme-Linked Immunosorbent Assay (ELISA)

For measurement of protein levels of inflammatory cytokines, ELISA kits for TNF-*α*, IL-1*β*, IL-6, and CINC-3 were purchased at R&D Systems (Abingdon, United Kingdom) and performed according to manufacturer's instructions.

### 2.6. Quantitative Real-Time Polymerase Chain Reaction (qRT-PCR)

All qRT-PCR materials were purchased at Roche (the Netherlands), unless otherwise stated. Hundred *µ*L of chloroform was added to heart homogenate in Tripure isolation reagent. Samples were then centrifuged for 15 minutes at 12000 RPM at 4°C. The supernatant was added to 250 *μ*L of 2-propanol and left untreated at room temperature for 10 minutes. After centrifugation for 10 minutes at 12000 RPM the supernatants were discarded and the pellet washed with 500 *μ*L of 75% ethanol. This mix was again centrifuged for 5 minutes at 12000 RPM. The pellet was dissolved in RNAse-free water and heated for 10 minutes at 60°C, after which all samples were kept at −80°C.

RNA concentration was measured on the Nanodrop 2000 (Thermo Scientific, the Netherlands). Complementary deoxyribonucleic acid (cDNA) synthesis was conducted with one microgram RNA using the Transcriptor First Strand cDNA Synthesis kit. One microliter cDNA was used in a total volume of 10 *µ*L PCR mix per reaction. Each mixture contained 10 *µ*M of primer pairs and 2x LightCycler 480 SYBR Green I Master. Real-time qPCR amplification was carried out using the LightCycler 480 instrument under the following conditions: preincubation at 95°C for 10 minutes, followed by 45 cycles of 95°C for 10 seconds, 60°C for 10 seconds, and 72°C for 15 seconds. The fluorescence measured per cycle of each sample was acquired. Primer sequences of genes for qRT-PCR were as follows: TNF-*α* (forward CTG GGA CAG TGA CCT GGA CT; reverse GCA CCT CAG GGA AGA GTC TG), IL-1*β* (forward GCC CAT CCT CTG TGA CTC AT; reverse AGG CCA CAG GTA TTT TGT CG), IL-6 (forward AGT TGC CTT GGG ACT GA; TCC ACG ATT TCC CAG AGA AC), CINC-3 (forward GTG CTA AGG CAT TGT GGT GTG T; reverse GCA ACA TCT TAT CAG TCC ATG GTT), and GAPDH (forward TGC CCC CAT GTT TGT GAT G; reverse GCT GAC AAT CTT GAG GGA GTT GT), which were purchased from Invitrogen (The Netherlands).

Raw data was exported as text file format and subsequently converted into an Excel sheet by using the program LC480Converter. The converted data was imported to the LinRegPCR program [12] and baseline correction was carried out, thus measuring fluorescence before amplification-specific fluorescence can be determined, consisting out of fluorescence from cDNA, primers, and unbound SYBR Green. Hereafter, individual PCR efficiency of each sample was obtained from the slope of the regression line fitted to a subset of baseline-corrected data points in the log linear phase [12]. Finally, the relative quantities of each target gene were calculated by normalization to the housekeeping gene GAPDH.

### 2.7. Sample Size Analysis and Statistical Analysis

Sample size analysis for each part of the study was based on results obtained from an earlier study [7] in which the primary endpoint was infarct size. The expected difference in mean was 18%, the standard deviation 12%, the power was 80%, and the type I error 0.05, resulting in a required number of rats per group being 8. Due to a suspected drop out of 20% due to surgical error, fatal arrhythmias or technological problems with staining, enzyme linked immunosorbent assay (ELISA) or polymerase chain reaction (PCR), we performed a total of 158 experiments, of which 22 dropped out.

Statistical analysis of infarct size experiments, biomarkers, and inflammatory protein levels was performed in GraphPad Prism (GraphPad Software, La Jolla, CA, USA) using one-way ANOVA with a Dunnet post hoc test comparing the control group against all other groups. Baseline hemodynamics were tested using one-way ANOVA with Tukey's post hoc test. For an overall effect on time and an intergroup difference in time, a two-way repeated measures ANOVA with Bonferroni correction for multiple testing was used. For inflammatory mRNA levels, a comparison between Sham and all other groups was tested by one-way ANOVA with Tukey's post hoc test. To compare control with helium intervention at the various time points of reperfusion (5, 15, and 30 minutes) a two-way repeated measures ANOVA with Bonferroni correction for multiple testing was done. For all analyses, *P* < 0.05 was considered significant.

## 3. Results

### 3.1. Hemodynamics

Baseline values of hemodynamic parameters (mean arterial pressures and heart rates) did not vary between groups. Overall, mean arterial pressure and heart rates in all groups were affected by time, as they slowly decreased during the experimental protocol (*P* < 0.05, not marked in table). Exposure of rats to any helium postconditioning protocols did not change mean arterial pressures or heart rates over time in comparison to control. For an overview of hemodynamics also see Table 1.

### 3.2. Infarct Size and Biomarkers LDH and TnT

**Figure 1:**
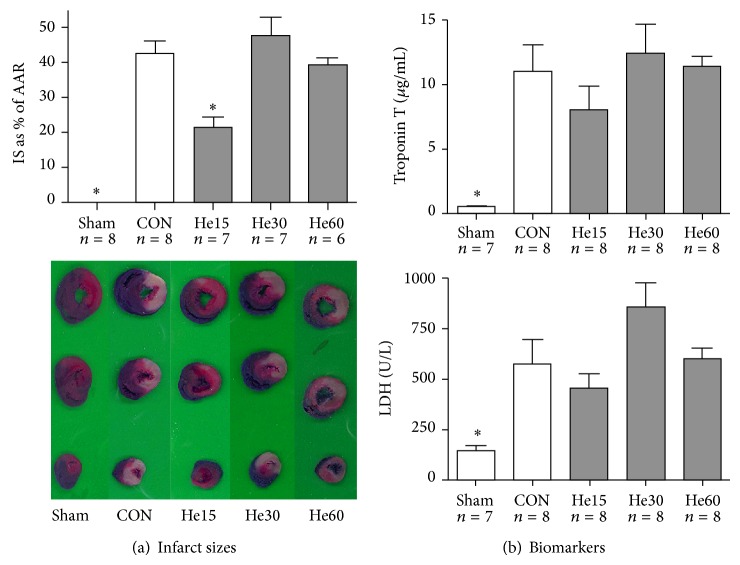
Infarct size and biomarkers after 120 minutes of reperfusion. Data are shown as mean ± S.E.M. ^*^ *P* < 0.05 versus CON. Amount of experiments in each group is shown below individual bars. (a) Infarct sizes as percentage of the area at risk. Underneath the graph, pictures of representative slices of myocardium are shown for each group. (b) Troponin T and LDH levels in the circulation after 120 minutes of reperfusion.

Fifteen minutes of helium reduce infarct size as percentage of the area at risk from 43% in control animals to 21%, whereas prolonged helium inhalation for 30 or 60 minutes during early reperfusion did not; also see Figure 1. There were no significant differences in area at risk as percentage of total ventricular tissue between groups. Analysis of biomarkers within each experimental group shows that LDH- and TnT-release in serum increases when reperfusion time increases (data not shown).  Figure 1(b) shows LDH and TnT-levels in the Sham, CON, He15, He30, and He60 groups after 120 minutes of reperfusion.

### 3.3. Protein Levels of CINC-3, IL-6, IL-1*β*, and TNF-*α*

**Figure 2:**
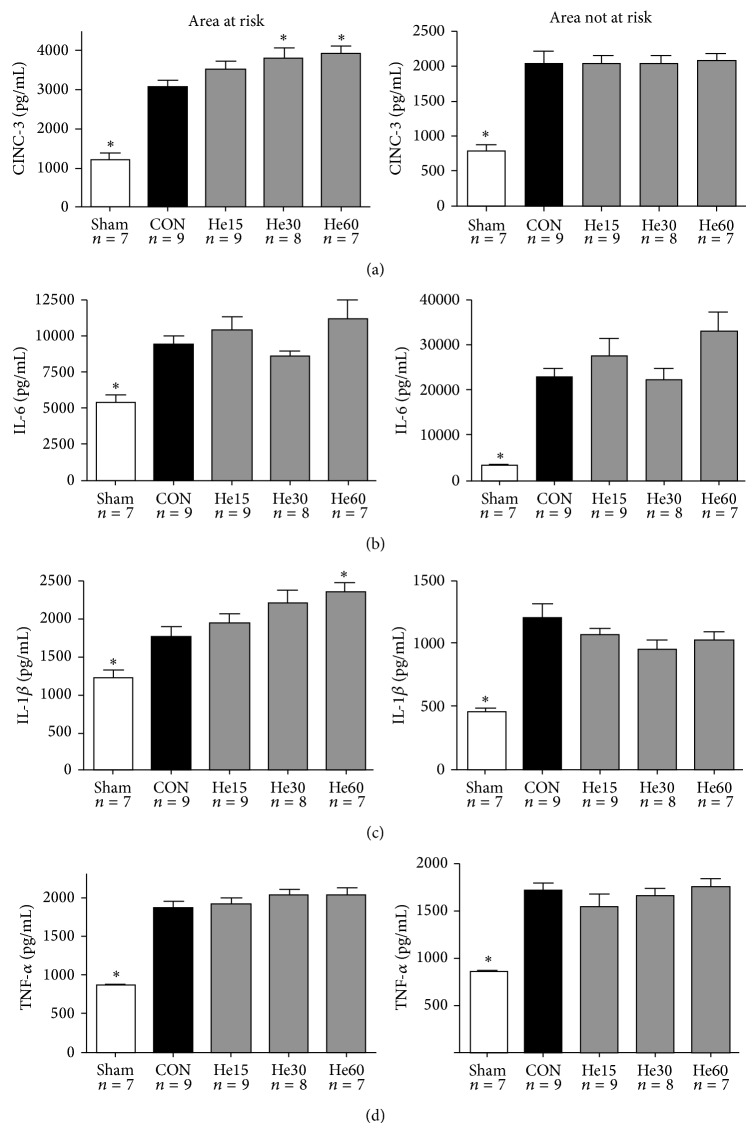
Protein levels of CINC-3 (a), IL-6 (b), IL-1*β* (c), and TNF-*α* (d). Protein levels are determined in myocardial tissue after completion of the experimental protocol: all animals underwent 15 min of stabilization, 25 min of ischemia, and 120 min of reperfusion, except for Sham animals. Sham animals were not exposed to ischemia and reperfusion. Animals receiving helium postconditioning received 15, 30 or 60 min of helium. All data are shown as mean ± S.E.M. Amount of experiments in each group is shown below individual bars. Groups were tested with one-way ANOVA plus a Dunnet post hoc test comparing the control group against all other groups; ^*^ *P* < 0.05, significant in comparison to CON.

In myocardial tissue exposed to I/R (AAR), CINC-3 protein levels were significantly higher in the He30 (3821 pg/mL) and He60 (3821 pg/mL) group in comparison to the CON (3097 pg/mL) group (*P* < 0.05). IL-1beta levels were significantly higher in the He60 (11787 pg/mL) group in comparison to the CON (6183 pg/mL) group. All cytokines were lower in the Sham group in comparison to the CON group. For protein levels also see Figure 2.

### 3.4. Messenger RNA Expression of CINC-3, IL-6, IL-1*β*, and TNF-*α*

**Figure 3:**
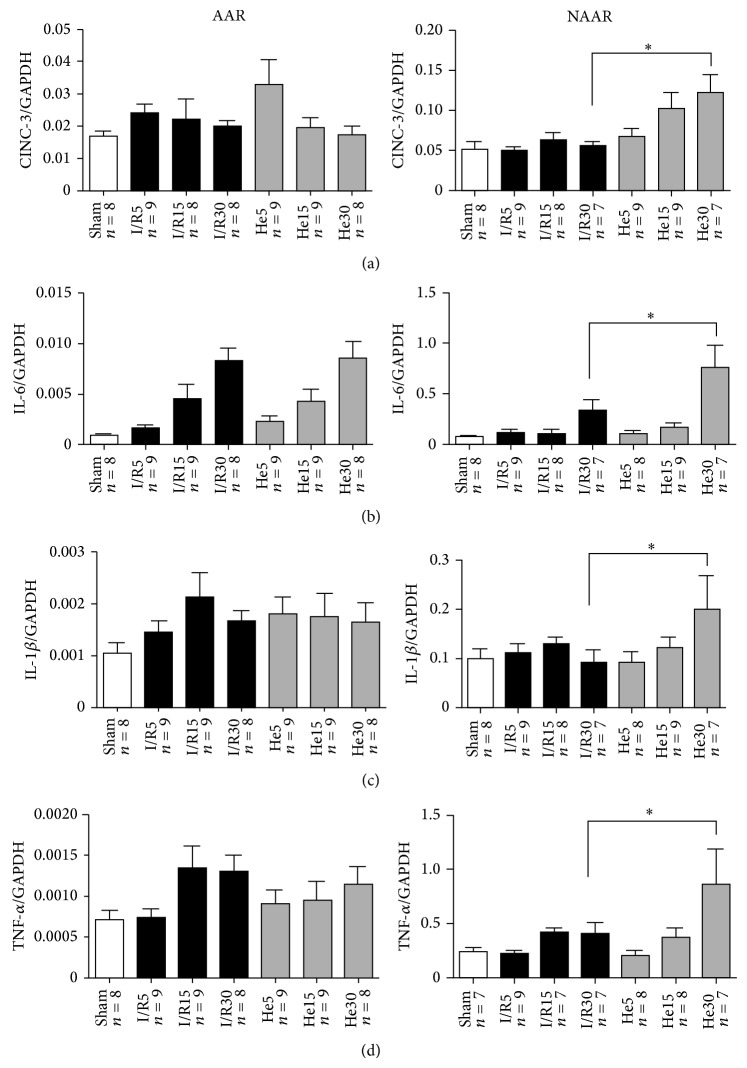
CINC-3 (a), IL-6 (b), IL-1*β* (c), and TNF-*α* (d) mRNA expression. Messenger RNA was analyzed in myocardial samples taken at the end of the experimental protocol. All animals underwent 15 min of stabilization and 25 min of ischemia (except for Sham) and 5, 15, or 30 min of reperfusion with or without helium. All data are shown as mean ± S.E.M. Amount of experiments in each group is shown below individual bars. To compare control (I/R) with helium intervention (I/R + He) at the various time points of reperfusion (5, 15, and 30 minutes), a two-way repeated measures ANOVA with Bonferroni correction for multiple testing was done. ^*^ *P* < 0.05 I/R in comparison to He.

In the AAR myocardium, IL-6 levels were significantly higher in the I/R30 (8.3 ∗ 10^−3^) and He30 (8.5 ∗ 10^−3^) group in comparison to the CON (9.7 ∗ 10^−4^) group, also see Figure 3. In NAAR myocardium, all measured targets (IL-6, TNF-*α*, IL-1*β*, and CINC-3) increased with the course of the reperfusion time. Additionally, a specific and significant difference between control and helium groups was found after 30 min of reperfusion (marked with ∗ in figure) for all inflammatory cytokines. Helium significantly increased the mRNA expression level of all cytokines at 30 minutes of reperfusion. These results can also be seen in Figure 3.

## 4. Discussion

In this study we show that infarct size reduction after helium postconditioning is determined by the duration of the postconditioning protocol; a short episode of helium postconditioning (15 min) reduces infarct size, whereas 30 and 60 min abrogate this protection. This is a relevant finding when helium-induced cardioprotection would be translated to clinical practice; apparently helium postconditioning is sensitive to the length of helium inhalation. Prolonged helium inhalation during ischemia and 180 min of reperfusion has shown not to reduce infarct size or extent of no-reflow in rabbits [13]. The negative effects of this study could also be caused by the timing of helium application, inhalation starting at the onset of the ischemic episode. In our study, it is striking that even a short application as 30 min is not protective; despite the general consensus on ischemic postconditioning stating it should comprise short stimuli [1]. The optimal duration of helium inhalation during reperfusion, that is, the definition of “short,” should be investigated, especially when the step from rat to human is being made. It seems clear though that helium postconditioning should always be administered at the onset of reperfusion, as delay will abrogate protection [14].

This study not only shows a reduction of infarct size after 15 min of helium but also shows the return of infarct sizes to control levels after 30 and 60 min of helium inhalation. In this* in vivo* setting and at this time point (120 min of reperfusion), prolonged helium application seems not to increase cellular damage. However, release of TnT and LDH into the circulation is not conclusive, as they show a trend comparable to infarct sizes but no significant difference. To our knowledge, no* in vivo* studies exist that report increased cellular damage after helium inhalation. However, one study presented increased cellular damage in human tubular kidney HK2 cells after exposure to 75% helium. The cells were exposed to three hours of oxygen glucose deprivation and incubated for 3 hours 75% helium 24 hours before the cytotoxic stimulus, which resulted in reduced cell viability in comparison to oxygen glucose deprivation alone [15]. A link between cell damage and increased immune activation arose from studies looking at cytotoxic effects of helium pneumoperitoneum in animal models of abdominal malignancy [16, 17]. Helium in this regard results in a reduced abdominal tumor spread and an increased immune activation.

Both* in vitro* and* in vivo* data suggest that helium can reduce cell damage when applied for short episodes [7, 8]. We hypothesized that a short episode of helium inhalation during early reperfusion leads to a reduction of tissue damage by a reduction of the deleterious sterile innate immune response, that is, reducing the hyperacute reactive oxygen species- and neutrophil-induced cytokine burst. The exact spatial and temporal function of the different components of the innate immune response are unknown, but new insights propose the “danger model” as a concept for the initiation of the immune burst during early reperfusion. This model suggests that immunity can be triggered by release of damage-associated molecular patterns (DAMPs) from cells in danger or stress (such as ischemia/reperfusion) [18]. The myocardium and myocardial endothelial cells then release cytokines and complement, which cause a toll like receptor-mediated influx of inflammatory cells and upregulation of cytokine mRNA expression and cytokine release [10, 19].

Interestingly, our study shows that the cardioprotective effects of helium were* not* accompanied by a reduction of inflammatory cytokines, neither at 15 or 120 min of reperfusion. The increase of inflammatory cytokine proteins is known to rise sharply within the first 12 hours after ischemia and returns back to normal within 48 hours [20]. We hypothesized that if helium postconditioning affects the innate immune system, an effect on protein levels of inflammatory cytokines should be found shortly after but not during the application of the protective stimulus. In AAR myocardium, IL-6 mRNA levels in the myocardium increased when reperfusion time increased: two-way ANOVA analysis showed that factor “time point” significantly affected outcome for this specific cytokine. It has to be noted that at 30 min of reperfusion, peak-IL-6 levels were probably not reached yet. Additionally, mRNA expression of all targets in NAAR myocardium after helium conditioning was mostly influenced by factor time as well and a “rise and fall” was not detected either. At 120 min of reperfusion, a “rise and fall” of protein levels of the aforementioned targets was not observed either. Possibly, the time points of analysis could have been too early to detect the final magnitude of the cytokine burst, which could explain the results. Additionally, levels of proinflammatory cytokines are increased in acute experimental models as a result of the stress of surgery. This is demonstrated in Figure 2, showing fairly high levels of all targets in Sham animals, both in AAR and in NAAR tissue.

Moreover, the actual amount of proinflammatory cytokines in the myocardial tissue is not only dependent on production by resident cells in the myocardial tissue itself but also dependent on production by circulating cells. This is reflected in the differences between AAR and NAAR myocardium. In AAR but not in NAAR myocardium, an increase in CINC-3 and IL-1*β* protein levels was found after 60 minutes of helium. Possibly, only the stressed myocardium releases all of its endogenous cytokines, whereas the NAAR myocardium is not so much triggered to do this and in this tissue the role of circulating cell production of cytokines is relatively bigger.

A proinflammatory effect of helium inhalation can be found when looking at the mRNA data. In the NAAR myocardium a rise of mRNA level of all cytokines could be found after 30 min of helium application, showing that not only time of reperfusion (i.e., duration of a cell-damaging circumstance) but also addition of helium causes elevation of inflammatory cytokine mRNA levels. The separate testing of Sham groups to all other groups further supports our hypothesis that no differences between the Sham and control groups with longer reperfusion times (15 and 30 min) exist but that the group undergoing 30 min of reperfusion* under* helium inhalation significantly differs from the group not receiving helium inhalation. Although a causative relation between helium and upregulation of inflammatory markers cannot be claimed, it does suggest that longer inhalation of helium than the usual postconditioning protocol is associated with higher levels of mRNA of proinflammatory cytokines. This effect seems to be specifically present in myocardial tissue that was not exposed to ischemia/reperfusion.

Several limitations of this study have to be mentioned. First of all, peak levels of mRNA occur after 12 h of reperfusion [21] (being 50-fold in the myocardium at risk and 15-fold in the myocardium not at risk); but in the current study, mRNA levels were investigated after reperfusion times varying from 5 to 30 minutes. A transcriptional effect of helium postconditioning under circumstances of ischemia/reperfusion might, therefore, not be visible at this time point. However, as helium-induced postconditioning acts within a very short time window during reperfusion (only protective within the first 15 min), it is likely that significant cellular changes underlying these infarct size-sparing effects should originate within this time frame. Furthermore, transcription of mRNA precedes translation to proteins.

Secondly, measurement of biomarkers is most often used for longer reperfusion times, and true levels of biomarker release might only become apparent after a few hours of reperfusion [22]. In a comparable study in rats, TnT correlated with infarct size after 180 minutes of reperfusion [23]. The serum levels of injury markers such as LDH and TnT are dependent on the release by the cell and its cytoskeletal structure, and, therefore, it is possible that our 2 hours of reperfusion were too short. In general, TnT is released from myocytes at a slower rate than LDH [24]. Finally, the use of TnT as a marker of true cellular injury during ischemia/reperfusion might not be correct as TnT is also released during heavy exercise [25].

Thirdly, as isolated heart experiments have not been performed, the influence of circulating cells and their concomitant cytokine production cannot be ruled out. Hence, protein and mRNA levels in this study do not reflect production by resident cells in the myocardium only. The positive side of the* in vivo* model is the clinical resemblance; the clinical phenotype is always determined by the netto effect of all contributing elements.

It has to be noted that solely investigating mRNA and protein levels can never prove a causal relationship. The investigation of factors that directly evoke the immune response has not been done either. The DAMPs are at the beginning of this signaling cascade. In hepatic ischemia-reperfusion injury, liver dysfunctionality after hepatic surgery is being described as “a sterile inflammatory disorder,” which is evoked by DAMPs [26]. Of course, many factors contribute to cardiac ischemia/reperfusion injury. It is postulated that after occlusion of a coronary vessel, the “no reflow phenomenon” fires the sequel of events that happens afterwards [10]. In this process, microembolism, platelet activation, and neutrophil plugging lead to endothelial dysfunction, followed by a cytokine burst. This cytokine burst results in neutrophil activation, which is followed by extensive oxidative stress and concomitant endothelial damage, especially during reperfusion when normoxia is reestablished. We would like to emphasize the importance of oxidative stress and release of DAMPs in the vicious cycle of the sterile immune response and suggest this to be an area of future helium studies.

Finally, it cannot be ruled out that signaling through the innate immunity pathways simply does not play a role in helium postconditioning but that increases and decreases of cytokines just exist as an epiphenomenon. Apart from the aforementioned suggestions, further experiments aiming to investigate the innate immune response after helium postconditioning should also comprise longer reperfusion times, to await the full range in increase of protein and mRNA levels of proinflammatory cytokines.

## 5. Conclusion

In summary, 15 minutes of helium inhalation during early reperfusion protects the myocardium and reduces infarct size whereas a prolonged inhalation of helium (30 or 60 minutes) during early reperfusion is not protective. Helium-induced cardioprotection is not accompanied by a reduction of the hyperacute burst of inflammatory cytokines, but prolonged helium inhalation might contribute to a proinflammatory response.

## Figures and Tables

**Table 1:** Hemodynamics.

	Baseline 15 min	Ischemia 24 min	Reperfusion 15 min	Reperfusion 30 min	Reperfusion 60 min	Reperfusion 120 min
Mean AP (mmHg)						
Sham	124 ± 25	112 ± 21	95 ± 13	92 ± 23	100 ± 17	83 ± 27
CON	117 ± 23	94 ± 21	96 ± 17	87 ± 13	90 ± 21	78 ± 22
He15	109 ± 19	97 ± 32	84 ± 21	73 ± 11	87 ± 27	79 ± 28
He30	99 ± 23	78 ± 25	87 ± 24	82 ± 18	77 ± 19	62 ± 19
He60	102 ± 18	76 ± 32	71 ± 14	68 ± 17	72 ± 31	48 ± 11
Mean HR (beats/min)						
Sham	374 ± 39	387 ± 42	336 ± 51	332 ± 43	333 ± 47	312 ± 38
CON	347 ± 32	361 ± 29	351 ± 33	332 ± 27	325 ± 33	291 ± 30
He15	356 ± 57	381 ± 33	379 ± 23	369 ± 39	361 ± 43	329 ± 50
He30	347 ± 61	355 ± 41	354 ± 34	354 ± 20	327 ± 21	317 ± 37
He60	357 ± 50	363 ± 25	365 ± 32	355 ± 33	332 ± 32	294 ± 41

Hemodynamics (mean arterial pressure and heart rate) sampled after 15 minutes of baseline, 24 minutes of ischemia, and 15, 30, 60, and 120 minutes of reperfusion. Data are shown as mean ± SD. Mean AP = mean arterial pressure in mmHg; mean HR = mean heart rate in beats/minute.

## References

[1] Ovize M., Baxter G. F., di Lisa F. (2010). Postconditioning and protection from reperfusion injury: where do we stand: position paper from the Working Group of Cellular Biology of the Heart of the European Society of Cardiology. *Cardiovascular Research*.

[2] Girn H. R., Ahilathirunayagam S., Mavor A. I., Homer-Vanniasinkam S. (2007). Reperfusion syndrome: cellular mechanisms of microvascular dysfunction and potential therapeutic strategies. *Vascular and Endovascular Surgery*.

[3] Murry C. E., Jennings R. B., Reimer K. A. (1986). Preconditioning with ischemia: a delay of lethal cell injury in ischemic myocardium. *Circulation*.

[4] Zhao Z. Q., Corvera J. S., Halkos M. E. (2003). Inhibition of myocardial injury by ischemic postconditioning during reperfusion: comparison with ischemic preconditioning. *American Journal of Physiology. Heart and Circulatory Physiology*.

[5] Weber N. C., Preckel B., Schlack W. (2005). The effect of anaesthetics on the myocardium—new insights into myocardial protection. *European Journal of Anaesthesiology*.

[6] Huhn R., Heinen A., Weber N. C. (2009). Helium-induced early preconditioning and postconditioning are abolished in obese Zucker rats in vivo. *The Journal of Pharmacology and Experimental Therapeutics*.

[7] Oei G. T. M. L., Huhn R., Heinen A. (2012). Helium-induced cardioprotection of healthy and hypertensive rat myocardium in vivo. *European Journal of Pharmacology*.

[8] Oei G. T. M. L., Weber N. C., Hollmann M. W., Preckel B. (2010). Cellular effects of helium in different organs. *Anesthesiology*.

[9] Timmers L., Pasterkamp G., de Hoog V. C., Arslan F., Appelman Y., de Kleijn D. P. V. (2012). The innate immune response in reperfused myocardium. *Cardiovascular Research*.

[10] Arslan F., de Kleijn D. P., Pasterkamp G. (2011). Innate immune signaling in cardiac ischemia. *Nature Reviews Cardiology*.

[11] Toma O., Weber N. C., Wolter J. I., Obal D., Preckel B., Schlack W. (2004). Desflurane preconditioning induces time-dependent activation of protein kinase C epsilon and extracellular signal-regulated kinase 1 and 2 in the rat heart in vivo. *Anesthesiology*.

[12] Ramakers C., Ruijter J. M., Deprez R. H., Moorman A. F. M. (2003). Assumption-free analysis of quantitative real-time polymerase chain reaction (PCR) data. *Neuroscience Letters*.

[13] Hale S. L., VanDeripe D. R., Kloner R. A. (2014). Continuous heliox breathing and the extent of anatomic zone of no-reflow and necrosis following ischemia/reperfusion in the rabbit heart. *The Open Cardiovascular Medicine Journal*.

[14] Vinten-Johansen J. (2012). Postconditioning: If you snooze, you lose. *Cardiovascular Research*.

[15] Rizvi M., Jawad N., Li Y., Vizcaychipi M. P., Maze M., Ma D. (2010). Effect of noble gases on oxygen and glucose deprived injury in human tubular kidney cells. *Experimental Biology and Medicine*.

[16] Dähn S., Schwalbach P., Wöhleke F., Benner A., Kuntz C. (2003). Influence of different gases used for laparoscopy (helium, carbon dioxide, room air, xenon) on tumor volume, proliferation, and apoptosis. *Surgical Endoscopy and Other Interventional Techniques*.

[17] Dähn S., Schwalbach P., Maksan S., Wöhleke F., Benner A., Kuntz C. (2005). Influence of different gases used for laparoscopy (helium, carbon dioxide, room air, and xenon) on tumor volume, histomorphology, and leukocyte-tumor- endothelium interaction in intravital microscopy. *Surgical Endoscopy*.

[18] Matzinger P. (2002). The danger model: a renewed sense of self. *Science*.

[19] Arslan F., de Kleijn D. P. V., Timmers L., Doevendans P. A., Pasterkamp G. (2008). Bridging innate immunity and myocardial ischemia/reperfusion injury: the search for therapeutic targets. *Current Pharmaceutical Design*.

[20] Moro C., Jouan M.-G., Rakotovao A. (2007). Delayed expression of cytokines after reperfused myocardial infarction: possible trigger for cardiac dysfunction and ventricular remodeling. *American Journal of Physiology: Heart and Circulatory Physiology*.

[21] Deten A., Volz H. C., Driest W., Zimmer H.-G. (2002). Cardiac cytokine expression is upregulated in the acute phase after myocardial infarction. Experimental studies in rats. *Cardiovascular Research*.

[22] Mersmann J., Latsch K., Habeck K., Zacharowski K. (2011). Measure for measure-determination of infarct size in murine models of myocardial ischemia and reperfusion: a systematic review. *Shock*.

[23] Xiong J., Wang Q., Xue F.-S. (2011). Comparison of cardioprotective and anti-inflammatory effects of ischemia pre- and postconditioning in rats with myocardial ischemia-reperfusion injury. *Inflammation Research*.

[24] Li L., Hessel M., van der Valk L., Bax M., van der Linden I., van der Laarse A. (2004). Partial and delayed release of troponin-1 compared with the release of lactate dehydrogenase from necrotic cardiomyocytes. *Pflügers Archiv*.

[25] Nie J., Close G., George K. P., Tong T. K., Shi Q. (2010). Temporal association of elevations in serum cardiac troponin T and myocardial oxidative stress after prolonged exercise in rats. *European Journal of Applied Physiology*.

[26] van Golen R. F., Reiniers M. J., Olthof P. B., van Gulik T. M., Heger M. (2013). Sterile inflammation in hepatic ischemia/reperfusion injury: present concepts and potential therapeutics. *Journal of Gastroenterology and Hepatology*.

